# The moderating role of social support on depression and anxiety for gastric cancer patients and their family caregivers

**DOI:** 10.1371/journal.pone.0189808

**Published:** 2017-12-28

**Authors:** Ansuk Jeong, Ji Yeong An

**Affiliations:** 1 Assistant Professor, Department of Psychology, The University of Utah Asia Campus, Incheon, South Korea; 2 Associate Professor, Department of Surgery, Samsung Medical Center, Sungkyunkwan University School of Medicine, Seoul, South Korea, Department of Surgery, Yonsei University College of Medicine, Seoul, South Korea; Harvard Medical School, UNITED STATES

## Abstract

**Background:**

There is a consensus that cancer care should go beyond physical care as cancer patients and their family caregivers experience psychological burden, financial difficulty, as well as social relation issues. The current study aimed to investigate the moderating impact of social support on depression and anxiety of cancer patients and their family caregivers.

**Methods:**

Gastric cancer patients and their family caregivers who visited a university medical center in Seoul were approached for participation in the study. Fifty-two pairs of adult patients and caregivers participated in the study. Along with demographic information and the physical condition of the patients, such as pre-operation cancer stage and the type of gastrectomy, social support, depression, and anxiety were measured for patients and caregivers, respectively.

**Results:**

In the first round of analysis, patients’ depression was associated with age, while patients’ anxiety was related to income. On the other hand, caregivers’ depression was not associated with patients’ health and living arrangement. In the second round of analysis to examine the moderating effect of social support, patients’ income and social support were related to depression and anxiety, but the interaction of income and social support was only observed for anxiety. For caregivers, no interaction effects were found. Social support decreased the negative effects of low income status on the patients.

**Conclusion:**

While the income of the families with cancer cannot be adjusted in the short-term, their experience of social support can be managed by a proper support system. Diverse implications in medical settings are discussed.

## Introduction

Cancer has been a leading cause of death across continents. 14 million new cases and 8.2 million deaths were related to cancer worldwide [1]. Five-year disease free survival varies by type of cancer, with an estimated rate of 74.4% for gastric cancer in South Korea (2010~2014) [2]. Given the increase in survival for cancer patients, psychosocial factors can have a significant impact on patients as well as their caregivers. Since the introduction of psycho-oncology as a field of specialty in providing care for cancer patients, it has become more common to assess the psychosocial factors that govern the quality of life of cancer patients. A systematic review notes that social support can have an effect on a number of different health indexes including cardiovascular, endocrine, and immune function [3], and more specifically with disease progression among breast cancer patients [4].

Cancer patients with relatively good prognosis, however, might lose attention and support from those in their social network. This can leave the family caregivers as the sole source of support during the long process of treatment and survivorship. Caregivers of cancer patients are reported to experience physical and psychological difficulties and are vulnerable to developing depression due to the stressors related to caregiving [5–7].

In this regard, the current study aimed to investigate the moderating role of social support on psychological well-being of both cancer patients and family caregivers.

## Materials and methods

### Procedures

The Institutional Review Board of the research site, a university medical center in Seoul, South Korea, approved the study (IRB Number 4-2014-0861). The opportunity for research participation was advertised in the gastric cancer division of its cancer center. Adult gastric cancer patients who were 18 or older and able to communicate in both oral and written Korean language were recruited. In addition, patients were recruited at least one month after surgery; by that point in time patients start experiencing minimal pain and physical strength to the degree that they can perform daily activities with almost no difficulty, including eating regular foods. Patients who expressed their interest in the study met with the research nurse to complete the informed consent. Then the patient was administered the survey in a quiet room. It took about 20–35 minutes to complete the cross-sectional survey.

In order to recruit family caregivers, the family members who accompanied the patients to the clinic were included. Caregivers who were 18 or older and had no barrier to communicate in Korean were recruited. When the caregiver expressed their interest, the research nurse administered the survey after gaining the informed consent. Each participant of the dyads completed the survey individually in a quiet space in the medical center. Fifty-two dyads of adult patients and family caregivers, 104 persons in total, participated in the study. There were no withdrawals during the course of the survey. All individuals who volunteered to participate completed the survey.

### Measurements

#### Basic information

Demographic variables, including age, gender, marital status, education, religion, income, and living arrangement, were included in the patient and family caregiver questionnaires. These factors have been examined in previous studies of predictors of anxiety and/or depression among cancer patients [5–9]. To measure the physical condition of patients, pre-operation cancer stage and the type of gastrectomy were retrieved from the patients’ medical records, with the patients’ consent, by the medical doctor in the research team.

#### Social support

Duke-UNC Functional Social Support Questionnaire [10] was used to measure the perceived degree of social support. Participants were asked to rate the levels of 8 different types of support they received. Items of support include “people care what happened to me,” “love and affection,” “chances to talk to someone about problems at work or with my housework,” and “chances to talk to someone I trust about my personal and family problems”.

#### Depression and anxiety

The Hospital Anxiety and Depression Scale (HADS from below) was employed to measure the depression and anxiety of the participants [11]. Seven out of 14 items reflect depression; the other 7 items indicate anxiety. Total score of each subscale was used for depression (0 to 21) and anxiety (0 to 21) respectively in the analyses.

#### Analysis

Hierarchical multiple regression analyses were conducted to test the shared variance among the variables of interest. In the first round of analysis, we aimed to identify the significant predictors of depression and anxiety out of patients’ age, income, living arrangement, type of surgery, and cancer stage. In Model II, patients’ and caregivers’ social support were entered as predictors to see the effects on depression and anxiety of patients and caregivers, respectively.

The predictors shown to have significant main effects in the first round of analysis were selected to be entered in the second round of analysis to see the interaction effect with social support. In Model I, age and income were entered as predictors; in Model II, patients’ social support was entered as a predictor of patients’ depression and anxiety; and in Model III, the product terms of social support with age and income, respectively, were entered as predictors to investigate the moderating effect of social support.

## Results and discussion

As Table 1 shows, there were more male patients than female patients. Only 4 percent of the participants lived alone. More than two thirds of the participants finished at least high school. Most of the patients (82.7%) had partial gastrectomy as treatment. Regarding the caregivers, only thirty-six or thirty-seven out of fifty-two participants provided the information on their gender, age, and relation to the patient.

**Table 1:** Characteristics of the participants.

Items		*N*	Percent
<Patients>			
Gender	Male	30	57.7
	Female	22	42.3
Age	Mean	54.3	
	SD	12.1	
Income (USD/month)	Mean	3000	
	SD	1700	
Living arrangement	Alone	2	4.1
	With spouse	14	28.6
	With spouse and children	24	49.0
	With spouse and parents	3	6.1
	With spouse, children, and parents	6	12.2
Education	No schooling	1	1.9
	Primary school	3	5.8
	Middle school	7	13.5
	High school	22	42.3
	College/University	18	34.6
	Post-graduate	1	1.9
Cancer stage	Stage 1	35	67.3
	Stage 2	8	15.4
	Stage 3	9	17.3
Type of surgery	Partial gastrectomy	43	82.7
	Total gastrectomy	9	17.3
<Caregivers>			
Gender	Male	10	27.8
	Female	26	72.2
Age	Mean	45.0	
	SD	12.8	
Relation to the patient	Spouse	21	56.8
	Daughter	5	13.5
	Son	4	10.8
	Daughter-in-law	3	8.1
	Others	4	10.8

In the first round of analysis, Model I showed marginally significant effect of patients’ income on patients’ anxiety (Table 2). On the other hand, no predictors related to patients’ health or living status explained caregivers’ depression and anxiety in Model I (Table 3). However, when social support was entered in Model II, patients’ age had marginally significant effect on patients’ depression; patients’ income had significant predictability of patients’ anxiety. Also patients’ social support predicted patients’ anxiety, whereas caregivers’ social support explained both depression and anxiety of caregivers (Tables 2 and 3). In other words, there was no dyadic effect: patients’ social support neither predicted caregivers’ outcomes, nor did caregivers’ social support predict patients’ outcomes.

**Table 2:** Predictors of patient’s depression and anxiety.

Predictors	Patient’s Depression			
	Model I (*n* = 50)		Model II (*n* = 50)	
	*β* (SE)	*p* value	*β* (SE)	*p* value
Age	.387 (.267)	.157	.525 (.257)	.056
Income	-.498 (.019)	.101	-.427 (.018)	.141
Living arrangement	.125 (4.488)	.697	.205 (4.230)	.502
Kinds of surgery	-.219 (5.905)	.344	-.139 (6.660)	.592
Cancer stage	-.100 (3.108)	.668	-.125 (2.907)	.569
Patient’s social support			-.389 (.387)	.183
Caregiver’s social support			-.083 (.444)	.783
Predictors	Patient’s Anxiety			
	Model I (*n* = 48)		Model II (*n* = 48)	
	*β* (SE)	*p* value	*β* (SE)	*p* value
Age	.021 (.108)	.937	.273 (.079)	.170
Income	-.523 (.007)	.069	-.444 (.005)	.037
Living arrangement	.160 (1.446)	.592	.404 (1.049)	.078
Kinds of surgery	-.373 (2.309)	.105	-.244 (1.905)	.195
Cancer stage	-.024 (1.207)	.918	-.085 (.850)	.600
Patient’s social support			-.688 (.114)	.005
Caregiver’s social support			-.050 (.137)	.831

**Table 3:** Predictors of Caregiver’s depression and anxiety.

Predictors	Caregiver’s Depression			
	Model I (*n* = 43)		Model II (*n* = 43)	
	*β* (SE)	*p* value	*β* (SE)	*p* value
Age	.144 (.217)	.566	.207 (.202)	.379
Income	-.408 (.014)	.122	-.323 (.012)	.173
Living arrangement	.106 (2.925)	.709	.246 (2.699)	.354
Kinds of surgery	.000 (4.418)	.999	-.166 (4.151)	.407
Cancer stage	.246 (2.400)	.261	.133 (2.171)	.497
Patient’s social support			.245 (.246)	.271
Caregiver’s social support			-.618 (.293)	.017
Predictors	Caregiver’s Anxiety			
	Model I (*n* = 39)		Model II (*n* = 39)	
	*β* (SE)	*p* value	*β* (SE)	*p* value
Age	.178 (.088)	.465	.304 (.077)	.163
Income	-.332 (.006)	.188	-.217 (.005)	.305
Living arrangement	.034 (1.190)	.901	.227 (1.030)	.346
Kinds of surgery	.291 (1.797)	.165	.140 (1.584)	.442
Cancer stage	.152 (.976)	.467	.034 (.829)	.849
Patient’s social support			.082 (.094)	.680
Caregiver’s social support			-.634 (.112)	.008

We proceeded to investigate the main effects of age, income, and social support, as well as their interaction effects. In the second round of hierarchical multiple regression analyses, patients’ depression was explained by patients’ income and patients’ anxiety was explained by income, social support, and their interaction (Table 4). For caregivers’ outcomes, no predictors related to patients’ status or caregivers’ social support had significant predicting power (Table 5).

**Table 4:** Moderating effects of social support on patients’ depression and anxiety.

Predictors	Patient’s Depression					
	Model I (*n* = 50)		Model II (*n* = 50)		Model III (*n* = 50)	
	*β* (SE)	*p* value	*β* (SE)	*p* value	*β* (SE)	*p* value
Age	.105 (.149)	.534	.122 (.141)	.449	-.649(.900)	.526
Income	-.394 (.009)	.026	-.342 (.008)	.041	-2.105 (.049)	.038
Patient’s social support			-.337 (.227)	.042	-1.625 (1.733)	.189
Age*Social support					1.067 (.027)	.463
Income*Social support					1.925 (.002)	.074
Predictors	Patient’s Anxiety					
	Model I (*n* = 48)		Model II (*n* = 48)		Model III (*n* = 48)	
	*β* (SE)	*p* value			*β* (SE)	*p* value
Age	-.197 (.052)	.232	-.217 (.042)	.106	-1.445 (.244)	.067
Income	-.409 (.003)	.016	-.290 (.003)	.037	-2.439 (.014)	.002
Patient’s social support			-.566 (.069)	.000	-2.432 (.475)	.010
Age*Social support					1.607 (.007)	.117
Income*Social support					2.397 (.000)	.005

**Table 5:** Moderating effects of social support on Caregivers’ depression and anxiety.

Predictors	Caregiver’s Depression					
	Model I (*n* = 43)		Model II (*n* = 43)		Model III (*n* = 43)	
	*β* (SE)	*p* value	*β* (SE)	*p* value	*β* (SE)	*p* value
Age	.114 (.144)	.531	.068 (.140)	.698	-.183 (.699)	.836
Income	-.552 (.010)	.006	-.400 (.011)	.052	-.777 (.038)	.258
Caregiver’s social support			-.335 (.253)	.102	-.766 (1.281)	.450
Age*Social support					.353 (.022)	.764
Income*Social support					.481 (.001)	.558
Predictors	Caregiver’s Anxiety					
	Model I (*n* = 39)		Model II (*n* = 39)		Model III (*n* = 39)	
	*β* (SE)	*p* value			*β* (SE)	*p* value
Age	.236 (.055)	.173	.181 (.051)	.254	1.101 (.243)	.153
Income	-.589 (.004)	.002	-.407 (.004)	.027	-.675 (.013)	.248
Caregiver’s social support			-.401 (.092)	.031	.476 (.446)	.579
Age*Social support					-1.247 (.008)	.222
Income*Social support					.336 (.000)	.630

**Fig 1:**
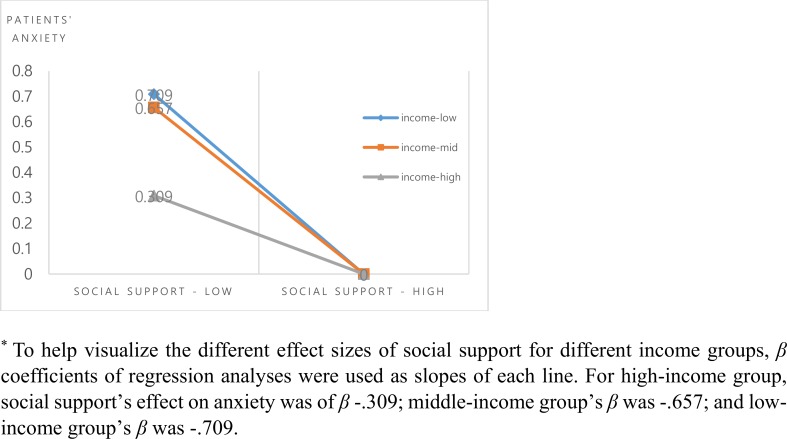
Patients’ social support and anxiety by income group.

The interaction effect of patients’ income and social support on patients’ anxiety was found (Fig 1). When the participants were grouped into three different income groups (low, middle, and high tertiles), the effect of social support was highest for the low-income group, followed by the middle-income group and high-income group. In other words, the impact of social support was higher for those in low-income group than for those in the high-income group.

## Conclusions

### Summary of results

In sum, the predictors that explained patients’ depression and anxiety were different from those that explained caregivers’ depression and anxiety. There were tendencies that older patients and patients with less income felt more depressed; and that patients with less income and patients with less social support felt more anxious. For caregivers, only caregivers’ social support explained their depression and anxiety: caregivers with less social support felt more depressed and anxious.

With respect to the interaction effect of social support and patients’ status, only patients’ anxiety was significantly explained by the interaction of patients’ income and their social support (for patients’ depression the interaction was marginally significant). More specifically, patients with less income was influenced more strongly by their social support or the lack thereof. By the same token, patients with more social support could be influenced less strongly by their income or the lack thereof.

### Implications

It is well documented that people in low income households have higher likelihood to experience depression and anxiety, generally speaking [12–13]. For cancer patients, who are vulnerable to external stimuli because of their medical condition, in particular, the lack of monetary resources can pose a serious stress. By the same token, any support provided for the families struck by cancer can be helpful, and, as a matter of fact, it was stated so by cancer patients and caregivers alike [14].

On the other hand, management of financial status of all families in a society might need plans on a larger scheme. While cancer patients go through psychological hardships, along with the physical pain and difficulties, social support provided by their family and friends can have rather direct effects. Social support can buffer the psychological burden of the lack of monetary resources in the treatment process. Notwithstanding the positive effects that social support can have, cancer patients and caregivers are reluctant to seek support from others because of the perceived stigma related to cancer [14]. Therefore, education of family members and community members about cancer and its psychological impact could enhance positive communication and promote the natural flow of social support, which in turn can help reduce patients’ anxiety and depression [15].

Similarly, methods to educate medical professionals to provide support to their patients could potentially promote the well-being of everyone involved as well. Even though we know that discussions about the illness, prognosis, plausible treatment plans between patient and the medical team enhance the psychological well-being of cancer patients [16], it is typically not enacted in the education curriculum for medical professionals. Learning how to talk to cancer patients and their family caregivers supportively should be essential in medical settings, particularly where the burden of cancer is high.

### Limitations and suggestions

With a set of 52 patient and caregiver dyads, it is hard to generalize the findings to other populations. The small number of participants might account for the lack of consistent results with other literature. For example, Given and colleagues’ study identified the most vulnerable family caregivers of cancer patients, according to their gender, age, employment status, and relationship to the patient [17]. However, the current study did not yield similar results, possibly due to limited statistical power.

Also the dataset was composed of gastric cancer only. Results cannot be generalized to other types of cancer, particularly if the prognosis is not as good, as in the case of lung or pancreatic cancer. For types of cancer that have lower survival rates, one of the biggest sources of concern for both patients and caregivers is the anxiety about relapse [14]. Furthermore, the dataset consisted of patients doing fairly well clinically. Thus the results might not be applicable to those with advanced cancer or in palliative care. Therefore, it is recommended to include various types of cancer, along with various stages of cancer, in the future studies.

### Conclusions

Despite limitations, the current study provides insight on the moderating role of social support when it comes to the effect of income on patient’s anxiety. Cancer patients will benefit from social support to overcome their anxiety related to the medical condition, more so with those of low income than of high income. This provides perspective for the efforts to mobilize resources to support cancer patients.

## Supporting information

S1 File The dataset of the current study. (SAV)
